# App-based multidisciplinary back pain treatment versus combined physiotherapy plus online education: a randomized controlled trial

**DOI:** 10.1038/s41746-019-0109-x

**Published:** 2019-05-03

**Authors:** Thomas R. Toelle, Daniel A. Utpadel-Fischler, Katharina-Kristina Haas, Janosch A. Priebe

**Affiliations:** 1 Technical University of Munich, School of Medicine, Department of Neurology, Munich, Germany; 2 Technical University of Munich, School of Medicine, Center of Interdisciplinary Pain Medicine, Munich, Germany; 3 Technical University of Munich, School of Medicine, Rise-uP Project of Innovationsfonds, Munich, Germany

**Keywords:** Pain management, Computational biology and bioinformatics

## Abstract

Non-specific low back pain (LBP) is one of the leading causes of global disability. Multidisciplinary pain treatment (MPT) programs comprising educational, physical, and psychological interventions have shown positive treatment effects on LBP. Nonetheless, such programs are costly and treatment opportunities are often limited to specialized medical centers. mHealth and other digital interventions may be a promising method to successfully support patient self-management in LBP. To address these issues, we investigated the clinical effects of a multidisciplinary mHealth back pain App (Kaia App) in a randomized controlled trial (registered at German Clinical Trials Register under DRKS00016329). One-hundred one adult patients with non-specific LBP from 6 weeks to 1 year were randomly assigned to an intervention group or a control group. In the intervention group, the Kaia App was provided for 3 months. Control treatment consisted of six individual physiotherapy sessions over 6 weeks and high-quality online education. The primary outcome, pain intensity, was assessed at 12-week follow-up on an 11-point numeric rating scale (NRS). Our per-protocol analysis showed no significant differences between the groups at baseline (Kaia App group: *M* = 5.10 (SD = 1.07) vs. control group: *M* = 5.41 (SD = 1.15). At 12-week follow-up the Kaia App group reported significantly lower pain intensity (*M* = 2.70 (SD = 1.51)) compared to the control group (*M* = 3.40 (SD = 1.63)). Our results indicate that the Kaia App as a multidisciplinary back pain app is an effective treatment in LBP patients and is superior to physiotherapy in combination with online education.

## Introduction

Non-specific low back pain (LBP), i.e., LBP without a known specific pathoanatomical cause, is a leading contributor to disease burden and disability worldwide, affecting people of all ages.^1,2^ More than 85% of patients presenting with LBP in primary care settings suffer from non-specific LBP.^3,4^ LBP exerts a tremendous socioeconomic impact, e.g., with costs in the United States exceeding $100 billion per year.^5^ Importantly, the majority of patients (65%) still complain of persistent pain one year after onset.^6^ It is widely accepted that LBP is a multidimensional condition characterized by an interaction of physical, psychological and social influences,^7,8^ referred to as the biopsychosocial disease model. Multidisciplinary pain treatment (MPT) programs focusing on physical, psychological, and educational interventions have proved effective in the treatment of subacute and chronic LBP with positive effects on pain intensity and disability.^7,8^ However, MPT programs are expensive and usually limited to specialized medical centers with waiting lists for admission.^9^

Digital interventions and the practice of medical and public health through the use of mobile devices (mHealth) has introduced new opportunities in health care.^10,11^ They have been proposed as a promising mode of delivery for self-management support programs and have been shown to effectively improve chronic pain.^12,13^ Nonetheless, systematic reviews found only weak evidence for beneficial effects of digital interventions in LBP management.^14,15^ However, the analyzed data seemed quite heterogeneous across the included trials. This may have been the reason for the overall inconclusive results, prompting the need for further trials in this field.^16–18^

The Kaia App is an mHealth app adopting a comprehensive evidence-based MPT program for non-specific LBP in accordance with current international disease management guidelines.^19–22^ The objective of the present randomized controlled trial was to compare treatment effects of Kaia App to a control group of patients who received a therapy program consisting of physiotherapy guided by a certified therapist plus online patient education on various issues of back pain. We hypothesized that the patients who were treated with Kaia App would report lower pain intensity after a follow-up period of 12 weeks.

## Results

### Included patients

**Fig. 1:**
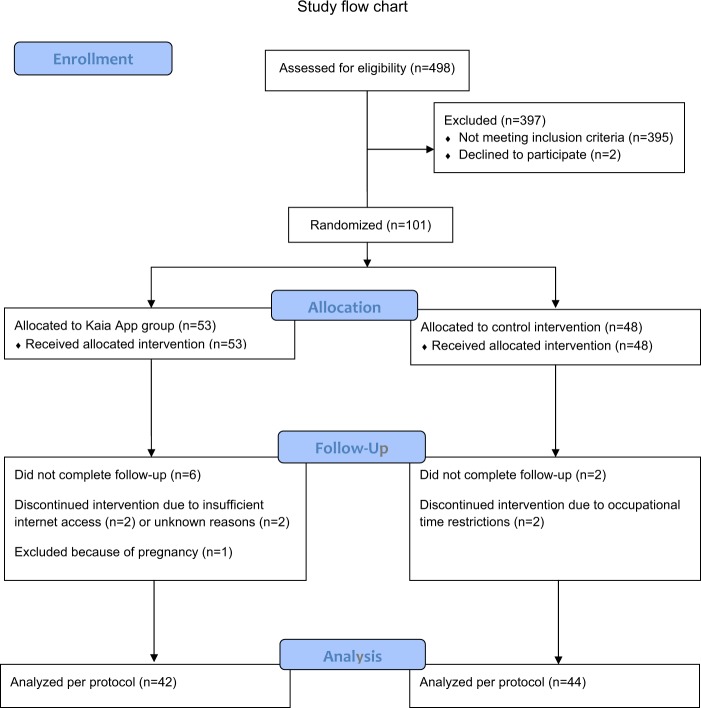
Study flow chart according to CONSORT

Recruitment was started on 1st August 2017. Last follow-up data were received on 30th October 2018. Four-hundred ninety-eight patients were screened. A total of 101 patients met the inclusion criteria and were enrolled (Fig. 1). Owing to administrative errors at the start of the trial, a deviation from the protocol occurred, resulting in a 53:48 distribution. The large difference between screened and included patients results from a significant misunderstanding from patients responding to the Facebook advertisement. Although stated clearly in the “Landingpage” that only patients with pain <1 year can be included, many patients with a life-long history of back pain responded and hoped to be included.

Reported reasons for drop-outs in the control group were occupational time restrictions and in the Kaia App group no sufficient internet access or unknown (two cases). With regard to attrition rate, six and one patients were lost to follow-up in the Kaia App and control group, respectively, providing no data at both follow-ups. One patient in the control group provided only 12-week follow-up data. Thus, follow-up rate at 6 weeks was 87.5 and 93.5%, and at 12 weeks 87.5% and 95.7% in the intervention and control group, respectively. Owing to administrative errors and patient withdrawal of consent during the study a total of 48 patients in the intervention group and 46 in the control group were included. There were no significant group differences regarding patient baseline characteristics (Table 1), nor regarding preceding and concomitant treatments, as reported by the patients as part of the questionnaires.

**Table 1:** Study baseline characteristics

Characteristic	Kaia App group (*N* = 48)	Physiotherapy plus online education group (*N* = 46)	*p*-value
Female	35 (72.9%)	31 (67.4%)	n.s.
Age (years)	41 (10.6)	43 (11.0)	n.s.
BMI (kg/m^2^)	24.4 (3.31)	25.4 (4.6)	n.s.
Education level			
Academic	27 (56.3%)	27 (58.7%)	n.s.
Duration of LBP (months)	7.2 (3.4)	6.7 (3.1)	n.s.
Chronic LBP (≥3 months)	39 (81.3%)	37 (80.4%)	n.s.
Physiotherapy^a^	1.11 (4.03)	1.33 (2.15)	n.s.

All data shown are mean values with standard deviations in parentheses, except for sex, education level, and chronic LBP (number and percentage). *p*-values are calculated by two-sided *t*-test or *χ*^2^-test

*n.s.* not significant

^a^Attended physiotherapy sessions in the 4 weeks prior to enrollment

### Primary outcome

**Fig. 2:**
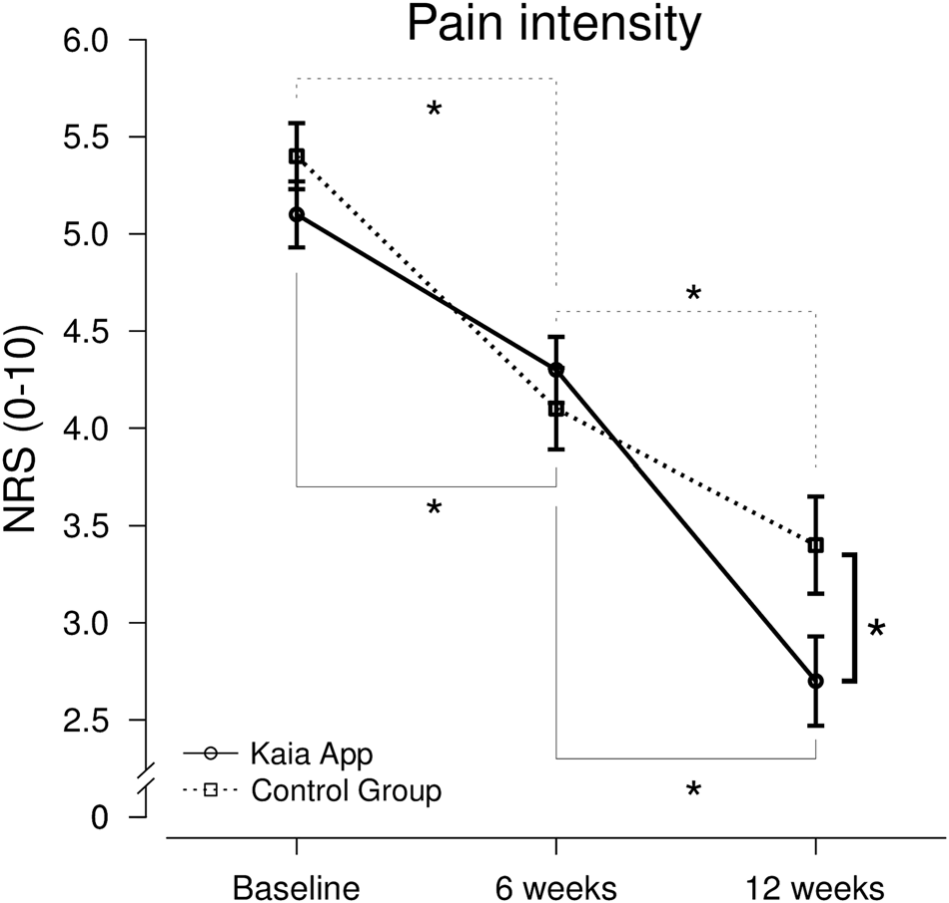
Mean of the pain intensity index over time separately for both groups, error bars represent SEM, Asterisk denotes Bonferroni-corrected *p*-values < 0.05 (*N*_Kaia App_ = 42; *N*_control group_ = 44; a two-factorial split-plot ANOVA was used as omnibus-test; post-hoc *t*-tests were used for within- and between-group comparisons)

Figure 2 illustrates the results of the analysis. The split-plot analysis of variance (ANOVA) with the between-factor group (Kaia App vs. physiotherapy + online education) and the within-factor measure point (baseline vs. 6 weeks vs. 12 weeks) revealed a significant main effect of measure point, *F*(2,168) = 31.38, *p* < 0.001, *η* = 0.492. Both groups reported a significant decrease in pain symptoms over time (baseline vs. 6 weeks and 6 weeks vs. 12 weeks) (all *p*’s < 0.01). Furthermore, a significant interaction of group and measure point, *F*(2,168) = 5.44, *p* = 0.031, *η* = 0.043, was found, which was driven by a significant lower pain intensity only after 12 weeks in the Kaia App group compared to the physiotherapy group, *t*(84) = 2.061, *p* = 0.021, while the between-group difference was not significant at baseline nor after 6 weeks (all *p*’s > 0.05). The main effect of group was not significant, *F*(1,84) = 1.54, *p* = 0.218, *η* = 0.018).

### Responder analysis

**Fig. 3:**
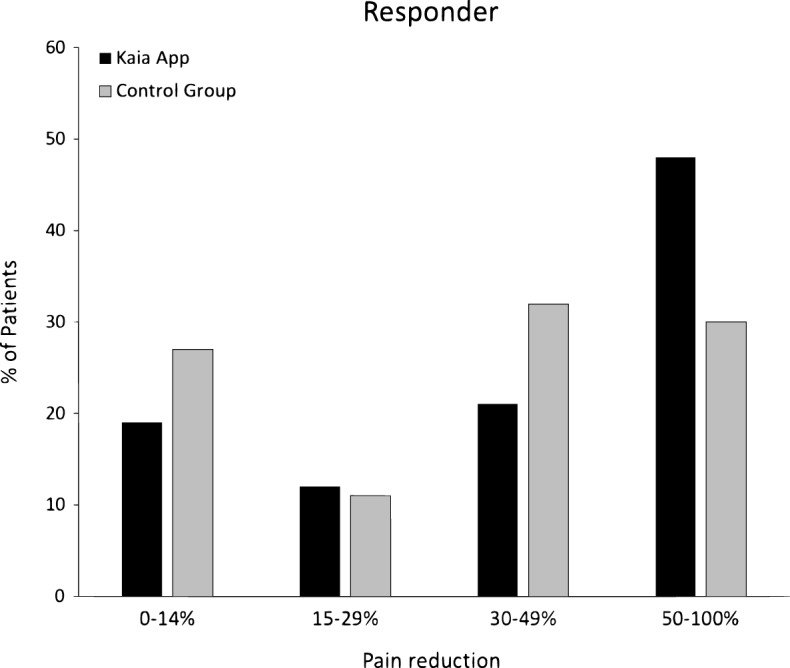
Responder analysis: Percentage (relative frequency) of patients regarding reduction of pain index from baseline to 12-week follow-up in percent separately for both groups (N_Kaia App_ = 42; N_control group_ = 44). *X*^2^-test did not reveal significance

Since the efficiency of the Kaia App was clearly shown, we conducted posteriori responder analysis for the primary outcome. For this purpose, Δ pain index scores were divided by the baseline value of each patient resulting in a percent score of pain relief. Next, we aggregated response rates below 15%, 15–29%, 30–49%, and above 50%. Hence, the distribution of patients between the four response sizes did not differ between groups, *X*^*2*^(3) = 3.33, *p* = 0.344; yet, descriptively, the Kaia App group is clearly overrepresented in the >50% group, whereas the control group has a peak in the 30–49% but also in the <15% group (Fig. 3). At 6-week follow-up three and four patients reported no current back pain in the intervention and control group, respectively. At 12-week follow-up 14 and 7 patients reported no current back pain in the intervention and control group, respectively.

Taken together, the analysis of our primary outcome revealed a substantially lower pain intensity in the Kaia App group compared to the physiotherapy plus online education group after 12 weeks.

### Secondary outcomes

No main effects (group, measure point) were observed with regard to functional ability and wellbeing measured by the Hannover Functional Ability Questionnaire (HFAQ) and VR-12 (all *p*’s > 0.05). Additionally, there were no substantial between-group differences in the Graded Chronic Pain Scale (GCPS) although there was a tendency of higher reduction in impairment in the Kaia App group. For physical and mental wellbeing (VR-12) there were significant main effects of measure point (mental: *F*(2,170) = 6.12, *p* = 0.003, *η* = 0.067; physical: *F*(2,170) = 48.54, *p* < 0.001, *η* = 0.363), indicating that both groups improved over time. Neither the main effect of group nor the interaction of group was significant (all *p*’s > 0.05). Table 2 provides an overview of the primary and secondary outcomes.

**Table 2:** Primary and secondary outcomes at baseline and 6- and 12-week follow-ups

Outcome	Kaia App group			PT^a^ plus online education group			
	Baseline (*N* = 42)	6 weeks (*N* = 42)	12 weeks (*N* = 42)	Baseline (*N* = 44)	6 weeks (*N* = 44)	12 weeks (*N* = 44)	*p*-value
Primary outcome							
Pain index^b^	5.10 (1.07)	4.33 (1.11)	2.70 (1.51)	5.41 (1.15)	4.09 (1.42)	3.40 (1.63)	0.021
Secondary outcomes							
HFAQ^c^	0.79 (0.14)	0.77 (0.17)	0.80 (0.12)	0.76 (0.15)	0.74 (0.12)	0.75 (0.23)	n.s.
GCPS^d^							n.s.
Grade I	18 (52.9%)	19 (55.9%)	27 (84.4%)	9 (27.3%)	19 (54.3%)	22 (62.9%)	
Grade II	13 (38.2%)3	14 (41.2%)	5 (15.6%)	17 (51.5%)	13 (37.1%)	12 (34.3%)	
Grade III	3 (8.8%)	1 (2.9%)	0	5 (15.2%)	3 (8.6%)	1 (2.9%)	
Grade IV	0	0	0	2 (6.1%)	0	0	
VR-12^e^							
MCS^f^	44.38 (10.08)	45.53 (7.39)	48.69 (8.38)	44.56 (9.29)	47.32 (8.25)	47.64 (8.11)	n.s.
PCS^g^	41.65 (8.00)	46.53 (9.01)	50.58 (6.86)	40.78 (8.18)	45.56 (8.78)	48.64 (8.22)	n.s.

All data shown are mean values with standard deviations in parentheses except for GCPS (number and percentage), *p*-values are calculated for between-group differences at 12 weeks by two-sided *t*-test or *χ*^2^-test

^a^Physiotherapy

^b^Pain index was calculated as the mean of current, maximum, and average pain intensity over the last 4 weeks

^c^Hannover Functional Ability Questionnaire

^d^Graded Chronic Pain Scale (calculated for subgroup of chronic LBP)

^e^Veterans RAND 12-Item Health Survey

^f^Mental component summary score

^g^Physical component summary score

### Kaia App activity

**Fig. 4:**
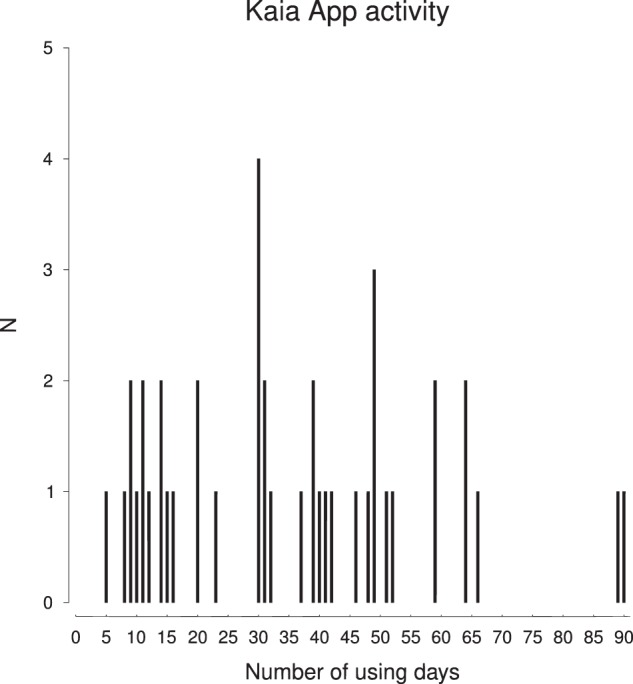
Overview of the frequency of app use in the app-group (*N* = 42)

Figure 4 provides an overview of the Kaia App use frequency. Within the observation period of 12 weeks, the Kaia App was used on average on *M* = 35 days (SD = 22 days). In order to examine, if frequency of Kaia App use is positively related to symptom improvement, bivariate correlations were computed between the pain index at baseline and after 12 weeks, as well as the Δ pain index. Interestingly, there was no significant correlation between Kaia App activity and Δ pain index (*r* = −0.005, *p* > 0.05). Additionally, the correlations between Kaia App activity and pain index at baseline and after 12 weeks were non-significant, although, there were positive tendencies for pain index at baseline (*r* = 0.244, *p* = 0.119) and after 12 weeks (*r* = 0.167, *p* = 0.290). In consequence, even though there was no relationship between Kaia App activity and symptom improvement, patients who reported a higher pain severity at baseline tended to use the app more frequently.

### Concomitant pain medication

Concerning concomitant pain medication there was no significant group difference at baseline nor at 6- or 12-week follow-ups, although a trend towards less intake of pain medication could be demonstrated in favor of the intervention group. Medication Quantification Scale (MQS) in the intervention and control group at baseline, 6- and 12-week follow-up *M* = 2.4 (SD = 3.31) vs. *M* = 2.8 (SD = 3.59), *p* = 0.56, *M* = 1.0 (SD = 2.07) vs. *M* = 1.7 (SD = 3.07), *p* = 0.22 and *M* = 0.9 (SD = 2.40) vs. *M* = 1.9 (SD = 3.01), *p* = 0.11, respectively.

### Adherence to physiotherapy and online education in control group

There was a high degree of adherence to the physical interventions. Of the possible 6 sessions, participants in the control group attended 89.8% sessions (mean 5.39, SD 1.22) sessions. In addition, no correlation between outcome measures and completed therapy sessions was observed. In terms of online education, after 6 weeks 62% and after 12 weeks 41% of the participants reported to have used the links. Sixty-two percent after 6 weeks and 41% after 12 weeks stated they considered the content useful.

### Adverse events

One patient in the intervention group was diagnosed with a lumbar disc herniation in the course of the running investigation, after his general practitioner (GP) had referred him for a routine lumbar magnetic resonance imaging (MRI) scan during the study. We believe that this was an incidental finding. Since the patient did not report new symptoms nor showed new signs in clinical investigations, a causal relation to exercising with the Kaia App seemed very unlikely. Apart from this case no other adverse events were observed.

## Discussion

To the best of our knowledge, this is the first randomized controlled trial to investigate treatment efficacy of an mHealth app digitalizing an MPT program in patients with subacute or chronic non-specific LBP in comparison to a control group receiving individual physiotherapy plus online education.

The enrolled population showed comparable clinical characteristics to other larger samples of LBP patients but in this trial there was a slightly more pronounced female preponderance and patients tended to be somewhat younger.^6,23–25^ Of note, most of the enrolled patients in this trial fulfilled the criteria for chronic LBP with 81.3% and 80.4% in terms of LBP that had been lasting for >3 months in the intervention and control group, respectively.

After 12 weeks the therapy program provided by Kaia App was superior with regard to the primary outcome: reported pain levels were significantly lower as compared to the control group (Fig. 2). As opposed to previous controlled trials which in most cases had no comparable or specific control intervention,^15^ we chose a control treatment taking into account existing clinical guidelines.^19–22^ In our opinion this is the remarkable strength in our study.

A Cochrane review found moderate to strong evidence from randomized controlled trials for the effectiveness of exercise therapy at reducing pain and functional limitations in the treatment of non-specific LBP.^26^ In addition to physiotherapy, patient education is advised as a first-line therapy for patients suffering from subacute or chronic LBP.^19^ Thus, the control group was treated with an evidence-based and guideline-oriented treatment. In fact, the recommended structured education of patients regarding back pain in current guidelines was more emphasized in the control group, than could be expected in standard care conditions, at least in Germany. We applied through an online program high-quality educational content accompanied by motivating messages.

The subsequent attenuation of pain reduction in the control group from 6-week to 12-week follow-up may be due to short term effects of physiotherapy. This might be explained by potential exercise discontinuation because of diminishing patient motivation upon completion of the 6-week physiotherapy course and factors emerging from an individual, face-to-face therapy setting with a personal therapist. Exact adherence data on exercise continuation of control patients after the 6-week physiotherapy course were not systematically collected.

In line with results of two retrospective analyses of Kaia App data^27,28^ the present randomized controlled trial demonstrated a significant reduction of reported pain levels. In another three-armed randomized controlled trial Irvine et al.^17^ compared efficacy of an online intervention (FitBack), providing education, behavioral strategies and instructional videos on specific physical exercises, with a waiting group (no intervention at all) and a group receiving online education only. Interestingly, after 4 months the FitBack group reported lower pain levels only compared to the waiting group, whereas FitBack was not superior to the online-education group. In contrast, in the present study, the Kaia App group showed superiority in spite of a guideline-oriented (physiotherapy + online education) intervention as control condition, which underlines the value of our app-based multidisciplinary approach.

Analysis of the number of completed active days with Kaia App at group level revealed a weak to moderate positive association between patient adherence and pain severity at baseline. This observation might suggest that a higher level of suffering consequently precipitates greater patient engagement. Otherwise, against our expectations no correlation between adherence and reduction of pain intensity was found. Nonetheless, to draw more reliable inferences about the determinants of app adherence the currently available evidence is insufficient. In particular, the two recent systematic reviews on digital interventions for LBP did not report any relevant data on adherence to the intervention.^14,15^

Remarkably, after evaluating the response rate of patients who reported at least 50% pain improvement compared to baseline, a distinct, though statistically not significant advantage was seen in the intervention group, suggesting that our results with respect to the primary outcome were also relevant from a clinical point of view (Fig. 3). Although the minimum clinically important differences (MCID) for chronic pain relief and LBP vary considerably and are still subject of dispute, we believe a >30% change from baseline may be considered a clinically meaningful improvement.^29–31^

With respect to functional ability, baseline scores revealed high functional ability in both groups. The missing over-time differences in functional ability may be due to this ceiling effect. In previous LBP trials, that included a functional assessment using the HFAQ, comparably lower mean scores, ranging from about 46 to 70, were reported, indicative of greater back-pain-related disability.^24,32–34^ However, the investigated patient populations were distinct from this study in terms of duration of back pain and other parameters.

At this stage, we cannot ascertain which of the treatment modules contained in the Kaia App account for the treatment effect. All modules may stand-alone or synergistically interact thus improving treatment outcome. Furthermore, by implementing reengagement emails and push notifications, treatment adherence may have been improved. However, our study design did not allow us to specifically assess the impact of these notifications and other relevant motivational factors.

Compared to “real-world” LBP patients, our study population was slightly younger and better educated, which may impact prevalence and adherence. However, the influence of educational level at least on prevalence is controversial. While some studies found an association of low educational status with an increased prevalence of LPB, other studies showed an inverse relationship between social status and the risk of LBP.^35^ Thus, we believe that this did not diminish the significance of our results.

The introduction of a selection bias by the recruitment via online advertising may be considered to weaken the significance of the results. Yet, this issue seems to be rather unproblematic since the Kaia App group did not show a substantially high affinity to technology (*M* = 2.53 on the TA-EG scale, which ranges from 1 to 5^36^). Upcoming results from another randomized controlled trial with the Kaia App (registration number: DRKS00015048) provide evidence for a substantial treatment success of Kaia in a large cohort of patients of older age, lower educational background and an exclusive inclusion process via GP-based recruitment (personal communication, publication in preparation).

Another limitation of our study is that although we found a substantially lower pain intensity after 12 weeks (predefined primary outcome) in the Kaia App group, the between-group difference in pain reduction was not significant. There was only a descriptive tendency in favor of the Kaia App group (Kaia App: *M* = −2.4; control group: *M* = −2,0; *p* > 0.05; *d* = 0.23). Yet, an a posteriori power analysis for the between-group comparison of the pain reduction (small effect) revealed an optimal sample size of >200 patients in each group. Since our a priori power analysis was based on our primary outcome, i.e., the pain level at 12-week follow-up, our study was clearly underpowered for this other analysis.

The current trial could not delineate the most relevant factor of the multidisciplinary Kaia App (education, physical training, mindfulness). This is due to the small sample size and the multiple potential combinations. However, we could observe that all three therapy modules were used by the participants to a comparable extent (physiotherapy: 1382×, mindfulness: 972×, education: 1085×). Addressing this important point more thoroughly would require a larger sample size, as well as analyses of a large retrospective data set. This is currently under investigation.

We opted not to include an additional waiting group in our trial due to ethical considerations (e.g., increasing risk of development and maintenance of chronic LBP when left untreated). Moreover, investigators were not blinded to subject group assignment for simplifying study design. Since we assessed patient-reported outcomes with standardized questionnaires, we think this is less relevant in terms of a potential bias.

Furthermore, as previously discussed, we did not systematically assess adherence to home exercises in the control group. Similarly, no process was implemented to verify if control group patients actually opened the provided education links.

In conclusion, in this randomized controlled trial, both groups experienced a significant pain reduction with a non-significant between-group difference. Despite that, we still argue that Kaia App is superior to physiotherapy plus education, as the Kaia App group reports significantly lower pain intensity than the control group, is safe and less expensive than the control intervention.

As a perspective, being rapidly available and easily accessible, the Kaia App might be especially useful early in the disease course for patients at high risk for developing chronic LBP. It would allow GPs to start effective low-cost treatment without delay. Thus, while waiting to be admitted to a specialized day clinic or hospital, patients could in the meantime be treated with Kaia App. Patients could also benefit from Kaia App to continue their pain self-management after completion and discharge from treatment, because a significant proportion develop recurrent pain and seek additional health care.^37^ It is accepted that there is a lack of sufficient LBP management in regular medical practice, potentially leading to sustained ongoing pain and potentially harmful non-evidenced based invasive procedures.^38,39^ Nevertheless, more studies will be needed to specifically investigate these issues and further our understanding of the applicability and full therapeutic potential of Kaia App and mHealth in general.

## Methods

### Trial design

We conducted a randomized controlled trial with two treatment groups while investigators were not blinded. Eligible patients were assigned to Kaia App or control group 1:1.

### Randomization

Patients were assigned to Kaia App or control group in an alternating fashion without allocation concealment, starting with the intervention group, i.e., the first enrolled patient was assigned to Kaia App group and the next patient to control group. Then, the sequence was continually iterated.

### Study population

Adult patients (aged 18–65 years) were eligible to participate in the study if they complained of non-specific low back pain (i.e., back pain without medical signs of a specific underlying cause such as tumor, infection, fracture, herniated disc or spinal stenosis) with a mean pain intensity of ≥ 4 on NRS over the preceding 2 weeks. Pain had to be ongoing for the last 6 weeks up to 12 months prior to inclusion. Participants were required to be fluent in German language. Only patients without experience with the Kaia App were included. Participants were excluded if they had a past medical history of a malignancy, spinal surgery or any other known serious medical condition, pregnancy, or if they exhibited any clinical signs of a specific underlying cause (“red flags”). A list of inclusion and exclusion criteria is available as supplementary methods.

Interested patients were submitted by GPs that had been recruited by the Center of Interdisciplinary Pain Medicine within the area of Munich, Germany, or via Facebook advertisements and announcement on the official website of the Center for Interdisciplinary Pain Medicine at the Klinikum rechts der Isar, Technische Universität München (https://www.mri.tum.de/schmerztherapie). After a short telephone interview to screen for inclusion and exclusion criteria, individuals were invited to a visit (t0) at our study-center for a personal assessment, including a physical examination by a medical doctor. Before enrollment, most of the patients had been seen by a GP because of their back pain.

### Intervention

Patients in the intervention group were provided access to Kaia App and encouraged by the clinical investigator to use the app on their smartphone or tablet at least four times a week throughout the study duration of 3 months. The Kaia App has been described in detail previously.^28^ In short, the Kaia App (Kaia Health Software GmbH, Munich, Germany) is a multiplatform mHealth app for iOS, Android, and native Web solutions. Kaia came to market September 2016 and is classified as a medical product class I. Patients were involved in the very first steps of development of Kaia App. In this context, patients (and in a second step also GPs) were invited for interviews and briefings to discuss feasibility. Moreover, semistructured interviews were conducted to assess adherence-related questions. Moreever, with the help of the checklist “ Mobile App Rating Scale” (MARS)^40^ patients needs and expectations were targeted. MARS is a brief and reliable tool to assess the quality of mHealth apps. In addition, criteria proposed by the German Federal Ministry of Education and Research (BMBF, https://ehealth-services.fokus.fraunhofer.de/BMG-APPS/) were incorporated in this process. Scenarios and potential strategies were further discussed in focus groups, including potential users.

**Fig. 5:**
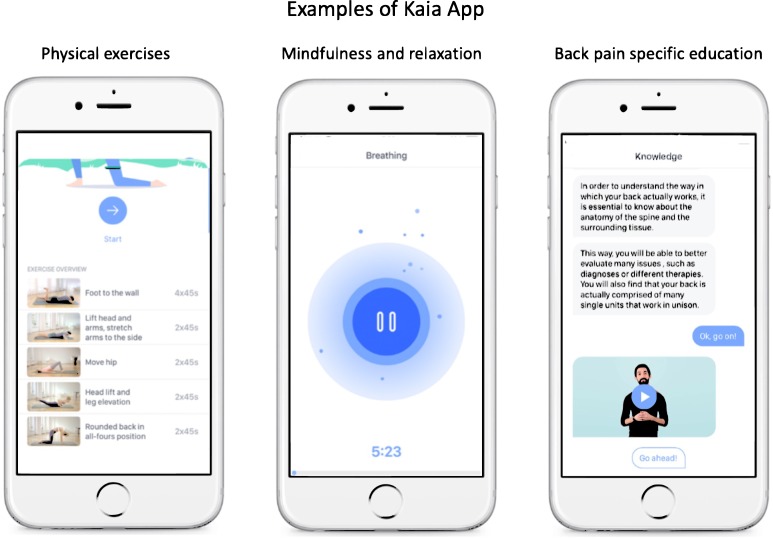
Examples of app content illustrating the three implemented main therapy modules

The Kaia App involves three therapy modules: (1) back pain-specific education, (2) physiotherapy/physical exercise, and (3) mindfulness and relaxation techniques (Fig. 5). Daily content consists of all three therapy modules. Content in the educational module covers a broad spectrum of general pain-related and back pain-specific education. There are over 30 different educational units in the Kaia App. Content is based on current international guidelines and standard textbooks in the field.^19–22,41,42^ Educational content was authored by board-certified physicians with relevant expertize in the field of back pain (i.e., neurology, orthopedic surgery, and pain medicine) and clinical psychologists with experience in pain psychotherapy. The single physiotherapeutic exercises and the individual composition of exercises for every user per day (up to five exercises) were designed by physiotherapists of the Center for Interdisciplinary Pain Medicine at the Klinikum rechts der Isar, Technische Universität München according to guidelines and curricula of the European Pain Federation (EFIC) and German Pain Society.^43^ Exercises are ranked depending on exercise difficulty and strain. Depending on user feedback, exercises are constantly adopted to the user’s fitness level. Mindfulness and relaxation techniques are recommended as an important part of multidisciplinary LBP treatment according to most guidelines.^19–22^ The Kaia App contains units of breathing techniques, body scan, visualization, and progressive muscle relaxation. The value of the various techniques is explained in the education module of the Kaia App. Mindfulness content is generally broadcasted as audio content only.

The content for an individual patient is compiled and updated from day to day (or upon each login) from a large background of exercises and skills archived in the Kaia App. Depending on the patient´s status of knowledge, practice, and progress this is adapted continually. Thus, exercise regimen and content are tailored to the individual patient. Each section is comprehensive as a stand-alone—there is no obligation to perform all 3 therapy modules in a single session. Pain levels are recorded using a 11-point numeric ratings scale (NRS; 0 = no pain, 10 = unbearable pain) at the end of each day of therapy in a pain diary.

National guidelines^19–22^ recommend physiotherapy for subacute and chronic back pain. Therefore, in the control group patients received six individual face-to-face sessions of standard physiotherapy once a week comprising physical exercises tailored to the individual symptoms and fitness level, as well as manual therapy. Minimal duration of the physiotherapy sessions was 20 min. The physiotherapy sessions were carried out by a certified physiotherapist at a local affiliated center for physiotherapy (therapiePUNKT GmbH). Furthermore, patients were encouraged to an active lifestyle and also to perform the physiotherapeutic exercises at home. In addition, links to a selection of medically oriented websites providing online resources for patient education about pathophysiology, diagnoses, treatment and self-management in LBP were sent to control group patients by the clinical investigators via email weekly, along with a brief motivating message (see supplementary methods for individual links). In total each control group patient received six emails during the trial.

### Outcome measures

Pre-specified measures for primary- and secondary outcomes were assessed by self-administered standardized questionnaires to be completed by the patients. At baseline, questionnaires were handed out by the clinical investigators (DUF, TRT) and completed via paper-pencil at our medical center on the day of inclusion. For 6- and 12-week follow-ups, questionnaires were sent to patients 1 week in advance via postal service. The completed questionnaires were resubmitted via postal service as well. Subsequently, data were entered electronically (DUF). Data sheets were double-checked by JAP.

We attempted to remind patients to complete the assessments via email requests and phone calls and to verify if they properly received our surveys when submission was overdue. Patients were not contacted further.

Pain intensity was assessed on a 11-points numeric ratings scale (NRS; 0 = no pain, 10 = unbearable pain) for current pain, as well as for maximum and average pain over the last 4 weeks. Pain intensity is a recommended core outcome domain for clinical trials in non-specific LBP and chronic pain.^44,45^ NRS is a reliable and valid method of rating pain intensity.^45,46^ In addition, a pain index was calculated as the mean of current, maximum and average pain intensity. The primary outcome was defined as the pain index at the 12-week follow-up.

Functional and behavioral measures were assessed at the 12-week follow-up as secondary outcomes. Functional ability was measured using the pain-specific German version of The HFAQ (FFbH-r “Funktionsfragebogen Hannover Rücken”). The HFAQ has been acknowledged to be well suited for measuring functional ability in patients with back pain.^47,48^ The questionnaire contains 12 questions about the ability to perform daily activities, rated on a Likert-scale. A functional ability score is calculated. Scores below 80% are considered as impairment, whereby scores around 70% equal a moderately, scores below 60% a severely limited function.

The GCPS has been widely used to collect data on both mental and physical aspects of chronic pain disorders, including chronic LBP.^49^ Thus, our analysis of GCPS was carried out in the subgroup of chronic LBP patients. A translated German version was tested in primary care back pain patients and found to be valid and reliable.^50^ Pain severity is graded into four hierarchical classes, according to simple scoring rules: Grade I, low disability-low intensity; Grade II, low disability-high intensity; Grade III, high disability-moderately limiting; and Grade IV, high disability-severely limiting.

Impairment of health-related quality of life was assessed using the German version of the Veterans RAND 12-Item Health Survey (VR-12).^51^ The VR-12 was derived from the Veterans RAND 36 Item Health Survey (VR-36) and contains 12 items relating to quality of life, including physical and mental health. Physical and Mental Health Component Scores are calculated.^52^ Both scores are *t*-transformed (*M* = 50, SD = 10). The VR-12 has been validated and is widely applied as a metric for tracking health-related quality of life

Data entered by Kaia App users as part of their self-test or app diaries stored on company servers in Frankfurt, Germany, were analyzed. Only pseudonymized data were extracted from the user database via reporting criteria and no personal data were submitted for scientific evaluation.

As part of the questionnaires, concomitant pain medication was assessed and quantified by the MQS.^53^

The number of completed physiotherapy sessions was assessed as part of the questionnaires at follow-up (“How many physiotherapy sessions did you attend?”). Regarding online education, patients were also asked at follow-up if they used the provided links meanwhile and if they found the content useful.

### Sample size

In order to determine the sample size, an a-priori power analysis was performed. For a two-way split-plot ANOVA with the between-factor group (Kaia App vs. control group) and the within-factor measure point (baseline—6 weeks follow-up—12 weeks follow-up), alpha-level of 5%, power of 80% and an expected medium effect size, a sample size of *N* = 82 is recommended. In order to compensate for drop-outs in the follow-up measurements with an estimated drop-out rate of 20–25%, and with respect to an unknown exact value of the effect size, >100 patients were considered as suitable. At the end, 101 patients were included.

### Statistical analysis

The primary outcome (pain index) as well as the secondary outcomes were subjected to separate two-way split-plot ANOVAs with the between-factor group (Kaia App vs. control group) and the within-factor measure point (baseline vs. 6 weeks vs. 12 weeks). In case of significant ANOVA-effects, Bonferroni-corrected post-hoc *t*-tests were run. Our main analysis referred to the patients who completed the questionnaires at each of the three measure points (baseline, 6 weeks, 12 weeks; *N* = 42 and 44 in Kaia App and control group, respectively; per-protocol analysis). Furthermore, an exploratory responder analysis was performed. We analyzed the frequency of patients in specific subgroups defined by response rate. Response rate was calculated as percentage reduction of pain index at 12-week follow-up in relation to the baseline: 0–14%, 15–29%, 30–49%, 50–100%. These cutoffs have been used previously.^54^ The 30 and 50% cutoffs have been used to define moderate and substantial benefits.^31^

GCPS data was analyzed by 3 separate *X*^2^–tests for each measure point. For comparing sample characteristics between groups, two-sided *t*-tests (metric variables) and *X*^2^–tests (categorial variables) were used. Next, the relationship between Kaia App activity and pain relief was analyzed in the Kaia App group. For this purpose, a difference score was calculated by subtracting the baseline pain index values from the values after 12 weeks (Δ) for each patient in the Kaia App group. Additionally, the number of days the Kaia app was used within the first 6 weeks and 12 weeks was calculated (“Kaia App activity”). Then, in order to quantify the relationship between Kaia App activity and pain relief bivariate correlations between the Δ-values and Kaia App activity were performed.

### Ethics and registration

The pre-specified study protocol was approved and registered by the Institutional Ethic Committee of the Medical Faculty of the Technische Universität München (registration number 170/17s) and in agreement with current data protection regulations. The trial is registered at DRKS (German Clinical Trials Register; WHO Primary Register) with No. DRKS00016329. Patients had to give written informed consent prior to enrollment.

## Supplementary information

Supplementary Methods

RepSum

## Data Availability

Data are available on request due to privacy or other restrictions.

## Supplementary information

**Supplementary Information** accompanies the paper on the *npj Digital Medicine* website (10.1038/s41746-019-0109-x).

## Code availability

All analyses were performed by SPSS IBM statistics version 25 and G*Power Version 3.1. Syntax files are available on request due to privacy or other restrictions.
