# Supplementary material for: App-based multidisciplinary back pain treatment versus combined physiotherapy plus online education: a randomized controlled trial
Source: NPJ Digit Med. 2019 May 3;2:34. doi: 10.1038/s41746-019-0109-x (PMC6550294; doi:10.1038/s41746-019-0109-x)
Supplement: Supplementary file 1 — Supplementary Methods [file 41746_2019_109_MOESM1_ESM.pdf]

Supplementary Methods

Inclusion criteria:

- Patients with lumbar back pain
- Age: 18-65y
- Pain duration: 6 weeks– 12 months
- Having a smartphone
- Average pain ≥ 4 NRS in the preceding 2 weeks

Exclusion criteria:

- Insufficient knowledge of German language
- Limited contractual capability
- Any malignant disorder in past medical history
- Radiculopathy with sensorimotor deficit
- Any spinal surgery in past medical history
- Any rheumatological disorder in past medical history
- Osteoporosis
- Infectious disorders of the spinal column
- Dementia
- Severe internal medicine disorders
- Severe neurological disorders
- Any known bone disorders
- Illicit drug use or alcohol abuse
- Pregnancy
- Prior subscription to Kaia App

Weekly educational links sent to patients in control group

Week 1:

https://www.tk.de/tk/sport/gesunder-ruecken/rueckenbeschwerden/20616

Week 2:

http://www.patienten-information.de/patientenleitlinien

http://www.patienten-information.de/kurzinformationen/ruecken/akuter-kreuzschmerz

http://www.patienten-information.de/kurzinformationen/ruecken/chronischerkreuzschmerz

Week 3:

https://nordost.aok.de/inhalt/rueckenschmerzen-2/

(meanwhile not available anymore)

Week 4:

http://www.br.de/gesundheitstag/rueckenschmerzen/rueckenproblemerueckenschmerzen-ruhe-bewegung-operation-100.html

Week 5:

https://www.dgss.org/patienteninformationen/schmerzerkrankungen/rueckenschmerz/

Week 6:

https://www.agr-ev.de/de/
